# An inflection point in global public health

**DOI:** 10.1186/s12992-022-00897-3

**Published:** 2022-12-05

**Authors:** Henry Greenberg

**Affiliations:** 1 Department of Epidemiology, Mailman School of Public Health, Columbia University, 722 W 168th St, New York, NY 10032 USA; 2 473 West End Ave. Apt 3-C, New York, NY 10024 USA

**Keywords:** Fiscal pandemic of good health, Primordial prevention, Obesity cascade, Science and economic development

## Abstract

Population health needs to pivot toward the primordial prevention of global chronic diseases, most specifically the disease cascade that runs from marketing to obesity to diabetes to its known complications. Medical sciences can now manage these diseases and prolong meaningful life, but can only do so at an enormous cost, a cost that will threaten societal stability everywhere. The fall in global fertility and the explosion in elderly populations will facilitate this fiscal pandemic attributable to good health. Risk factor mitigation, not effective for obesity, enhanced longevity but did not prevent chronic illness, only forestalled it. For public health, but not health practitioners, the risk factor era needs to be supplanted by a focus on public policy to alter public behavior via primordial prevention of the emergence of risk factors. And public health needs to lead that effort. The historical pathway to this present dilemma that linked science to economic development can be illuminated by the efforts of four scientists, Francis Bacon at the dawn of the seventeenth century, James Lind in the 18^th^ and Vannevar Bush and Abdel Omran in the 20^th^. This perspective introduces a near inevitability to the emergence of the current critical pivot point but also teaches that there is a powerful rationale to assume that dramatic and expensive changes will be coming and need be anticipated and planned for.

## Background

A path running through the past four centuries has led public health to a major inflection point. Public health has created powerful, transformative impacts on national and global health but has not forthrightly or sufficiently confronted new challenges that have emerged.

To remain relevant, the focus of global population health needs to pivot to chronic illnesses, more specifically, to their prevention using methodologies other than individual behavior change for risk factor mitigation. This rising threat is not due to failure of clinical care but to its success. The currency of the threat is not the disease but its cost. Advanced and many middle-income countries now and soon, all, are or will be facing a fiscal pandemic that will exert profound societal impacts unless confronted and tamed [1].

## Main text

Chronic diseases are not part of the DNA of public health. They came late to the party and, like an unwanted guest, are ignored. By the time they were recognized as a major problem, biological risk factor mitigation was well established and thought to be preventive. The success in reducing blood pressure, lowering cholesterol, and curtailing smoking lulled not just public health, but health economists into a form of seemingly perpetual somnolence. It is not that the problem is not recognized; it is. The data are everywhere and clear. One need go no further than the Global Burden of Disease summaries published biannually in Lancet [2]. In advanced economies, public health emphasizes risk factor mitigation and petitioning governments to change their ways, the former having established its role and reached a plateau in most advanced economies and the latter ineffective. Taxes on sugary drinks or fast food, with occasional exceptions, are tailored for revenue, not behavior change although do contribute to the national and public health budgets. In those countries dependent upon the donor community for health care funding, there is no concerted effort to push the donor community to step up and embrace chronic disease and hence no pressure to alter the curriculum in schools of public health [3]. The pot gets an occasional half-hearted stir, and the chefs walk away. Both groups, the donors and the public health practitioners, remain drowsy.

Before posing the arguments for a population health assault on chronic disease, three stalwart beacons of public health need to be dimmed, if not extinguished. First, HIV/AIDS has to be removed from the list of existential threats. To its enormous credit, global public health has trained cadres of sophisticated professionals in HIV/AIDS management around the world. While no cure or vaccine exists, this death-certain plague has been converted to a medically managed chronic illness compatible with a long life. Hence, there is no global career path for a western HIV/AIDS expert; public health should reduce and fade out its commitment to train them. Second, the era of risk factor mitigation programs aimed at individuals as a public health priority is coming to an end. It had a great run, created old people, but now must be left to practitioners and health ministries. In emerging economies, it needs to be pursued but folded into public health’s new focus on public policy (see below). The U.S. longevity had a dramatic spike upwards between the end of the 1960s into the early 1980s, likely in no small degree attributable to risk factor control. But the law of diminishing returns has set in and the low hanging fruit has been picked. Mitigation’s one flaw was the assumption that it was preventive when it only forestalled the arrival of clinical expression, a message increasing longevity has taught. The third new reality that needs to be accepted is that the one risk factor not curbed by any available interventions and increasing everywhere, obesity, cannot be treated and must be prevented. Even if the new medications can reverse severe obesity; even if the price can be brought down to comfortable levels globally; and even if the side effect profile is not off-putting, there would be little impact on prevention [4]. These drugs would keep gerbils on the wheel.

Primordial prevention [5] of obesity everywhere is the new challenge and trial for global public health. The obesity cascade— type 2 diabetes, heart disease, kidney failure, cancer, hip and knee replacement, and wheel chair dependency— is increasing in nearly every country on the planet [6]. The recent Global Burden of Disease report on cancer risk factors attributes 5% of cancers to obesity [7]. The employment, psychological, and emotional burdens are additional dimensions that carry heavy societal and fiscal burdens. India [8], much of Latin America [9], and small island countries in the Caribbean and Pacific Ocean [10, 11] are illustrative examples of the obesity crisis. The traditional risk factors—hypertension, hypercholesteremia, smoking—have been and will continue to be confronted by traditional public health interventions as well as clinical care systems, although they, too, are exacerbated by the commercial determinants of health [12, 13] that dominate the obesity epidemic.

### How did we get here?

Chronic diseases became the new top health and economic predators by two pathways. First, medical science developed the capability of incorporating preventive initiatives, most effectively drugs, into populations designed to dim the likelihood that a subset therein would develop in future time the targeted ailment. This is the most clear-cut instance in which the life span of people with multiple chronic diseases was extended by medical interventions; old people were created in plentiful numbers. Second, the technologically advanced medical interventions themselves became so sophisticated so as to permit life with the diseases to be tolerable, even comfortable, for decades. Old people get older, their sex lives improve, their golf scores fall, and they are happier, and importantly, they vote. During these “golden years”, garnering the rewards of a compression of morbidity [14], exacerbations of preventable chronic illness will require interventions, always expensive and often repetitive. Whether a bout of recurrent heart failure, a pacemaker battery change, the need for a coronary stent, an infection related to diabetes, or an obesity related hip replacement, all can be handled regardless of age and patients returned to a highly satisfying life style. See Bernie Sanders. In addition, considerable new effort is being directed at promoting healthy ageing in advanced economies [15], hence likely boosting longevity even higher. The fiscal burden of managing chronic illness is likely to increase, putting even greater pressure on the burden of prevention.

The social and intellectual history of how we got to the present predicament is a wondrously fascinating story. Its evolution suggests an inevitability about the arrival of the present situation. The tale has five chapters. There are many players on this historical stage, but four scientists stand out and can stand in for their myriad colleagues. It is their success and the success of health science, not failure, that has brought us to decision time. The four scientists are Francis Bacon (1561–1626); James Lind (1716–1794); Vannevar Bush (1880–1974); and Abdel Omran (1925–1999).

### Chapter 1: Elizabethan and stuart england

Sir Francis Bacon was of noble birth and rose to become the Lord Chancellor of England. He was a savant, a philosopher, a thinker, a writer, and a not-so-good scientist by most accounts. He was recognized in his life time as one of the dominant intellects of his age, but nearly unique among intellects of his age, or any other, he was well grounded in social, governmental, and political currents coursing through the land.

So what did Sir Francis do? He discovered no new scientific truths; he designed no clever tools or instruments; he discovered no new planets or comets; he wrote no new laws of nature. What he did was more important than new descriptions of nature’s design or clever widgets. He taught us how to think. While he built his arguments on the foundations conceived by others, as all great innovators do, he laid out the argument with clarity, precision, and detail. He developed what we now know as the scientific method. He argued for inductive reasoning, based on observation and experimentation. He decried the Aristotelian pattern of deductive reasoning as an inadequate and ineffective pathway to truth.

The second contribution flows from the first. Because his life was embedded in the political and social milieu of his times, his philosophical musings carried over into his vision of his secular world. He recognized that the outputs of the natural philosophers, the scientists of his age, offered benefits to the state. Hence, these tinkerers should be encouraged, not pilloried; valued, not scorned; supported, not shunned. His work became not only a foundational support for the Royal Society, established in 1660, but the scientific underpinning of the industrial revolution and the subsequent dominance of science in Western society. In his illuminating book, *The Culture of Growth,* economist Joel Moykr states “…Bacon’s heritage was nothing less than the cultural acceptance of the growth of useful knowledge as a critical ingredient of economic growth [16].” Bacon’s contribution to intellectual history also included creating a milieu in which the changes brought on by the industrial revolution were accepted.

One of his many famous aphorisms captures his contribution although only in the broadest outline, revealing no details, “Knowledge is Power.” Before Bacon power was the number of men under arms, the weight of gold in the treasury, the marital links to others with power, and/or the ability and capacity to carry out threats to people and places. Before the Baconian transformation, understanding inductive reasoning and knowing stuff hardly made anyone shudder.

### Chapter 2: The aftermath of world war II

July 5, 1945 witnessed a transformative change in American, and then global scientific enquiry. On that date Vannevar Bush sent to President Harry Truman a report requested months earlier by President Franklin Roosevelt. *Science, The Endless Frontier* [17] is now recognized as the founding document for an approach to the creation of new knowledge aimed at improving the lot of humankind and sustaining the self-perpetuating economic development needed to preserve the ever-improving way of life required for the maintenance of an open, free society.

Vannevar Bush, born into a very middle-class family in Massachusetts, became what one insider called the second most important person in the American government during World War II. Clearly gifted, and clearly aware of it, he excelled in many arenas, including his chosen field, electrical engineering. He was an innovative scientist, a founding contributor to what became the computer; an academic administrator; and a successful entrepreneur. Yet his fame rests on his career as science administrator during World War II.

In 1938 he interrupted his academic career at MIT and accepted the post as president of the Carnegie Institute of Washington, putting him in the maelstrom of policy development at the onset of the war. Building on his insight that the coordination between government and industry during the first world war was a significant failure, he persuaded Roosevelt to set up the National Defense Research Committee, which he chaired until 1941 when he became director of the Office of Scientific Research and Development, the president’s chief science advisor.

All his insights and management skills came to fruition in this position. He was able to get the suspicious military to trust civilian scientists who, under his guidance, solved problems the military needed solved. Radar, the proximity fuse, and the Manhattan project were the three jewels in the crown. Interestingly, considering his future contributions, universities played an important but not central role in these endeavors.

Both Bush and Roosevelt realized that science had the capacity to drive the economy. In both New England and the California Bay region (Stanford University), deliberations and postulations had emerged, but it took the stimulus of the war time experience and the recognition that the academic community would be a full-throated partner to jump-start the process [18]. *Science, The Endless Frontier* led directly to the creation of the National Science Foundation, established in 1950. Bush understood that the economy needed science to create new fields and new industries to engage the energies and support the desires of a modern society. He also understood that the enterprise needed support from three strong legs, government, academia, and industry. What is most fascinating about his report is that the breadth it describes goes beyond anything that was done during World War II. The universities played a modest role in the war. Now they are front and center, with vastly more authority. The research, strongly tilted toward biologic research and health, needed to be investigator-initiated, curiosity-driven, and given long term support, including indirect costs. The grantees needed well-defined independence, long- term funding, and the inclusion of both men and women. None of this was part of the World War II effort. These ideas obviously seeped into his thinking and were new and transformative components of his post-war plan. That he could expand his vision so far beyond what had earned him unending praise is truly remarkable. My only criticism of his report is that he did not give any credit to Francis Bacon.

### Chapter 3: The 1960s, with a remote prelude

In 1747, *HMS Salisbury* was in the English Channel enforcing a naval blockade during the War of Spanish Succession. Presumably having little engagement with enemy, its naval surgeon, James Lind found the time to carry out the first modern clinical trial. Scurvy was a scourge for navies, with mortality rates on one global circumnavigation reaching 70%. Citrus fruits had been thought to cure or ameliorate scurvy but this theory lacked sufficient supporting experience to become policy. Lind introduced a powerful new tool to confirm the supposition, the randomized clinical trial. He assigned 12 scorbutic sailors to one of six therapies that included cider, sulfuric acid (low dose), sea water, vinegar, “spicey paste,” and two oranges plus a lemon. The citrus pair was cured within a week; the recipients became nurses for the others as the stores of citrus had been depleted.

The first contemporary randomized clinical trial, designed and published by Bradford Austin Hill in 1948, showed that streptomycin was the first effective therapy for tuberculosis [19]. Randomized trials showing antibiotics better than placebo or not smoking safer than smoking need small numbers to show powerful advantages. The real power of the technique came in showing that modest changes in risk factors showed small but significant reductions in the occurrence of heart disease, stroke, or cancer. When applied to large populations, these modest interventions prevented, or rather forestalled, many clinical events and deaths. Numerous studies during the late twentieth century and beyond played a key role in the leaps in longevity seen in both advanced economies and mid-and low-income countries ([20] p 1231).

### Chapter 4: The epidemiologic transition

In 1971, a quiet, but respected journal, The Milbank Memorial Fund Quarterly, published a detailed article by an Egyptian-born epidemiologist, Abdel Omran then at the University of North Carolina, in which he described an epidemiologic transition from infectious disease to what he quaintly labeled, degenerative diseases [21]. The article has become a bright star in the firmament for epidemiology and an iconic publication for those focused on global chronic illness. A recent review written to commemorate the article’s 50^th^ anniversary finds much to fault, although most of its observations are valid [22]. Nonetheless, the current global data is even more tilted toward chronic illness than Omran described, the current COVID-19 pandemic notwithstanding.

In many ways Omran has become an accidental or inadvertent icon. His major focus, and what drove his career, was his commitment to reduce global fertility. He was an ardent champion of birth control and abortion liberation, arguing that if fertility rates fell, childhood mortality would plummet, children would be healthier, better educated, more productive, and live longer. This cascade of events would benefit any country on the planet but a corollary development would be an increase in “degenerative” diseases. While his cascade contains much truth, there are multiple other drivers of longevity than fertility, not the least of which is the intervention of medical science.

Omran inaugurated the discussion of the changing patterns of disease world-wide, forcing a recognition that chronic diseases are the major determinants of health in most advanced economies, and soon would be in all. At the very least, his article was an eye-opener, an invitation to look at global health patterns in a new way.

### Chapter 5: The synthesis—two pathways to fiscal armageddon

From Francis Bacon population health professionals (and everyone else) learned how to think; from James Lind how to organize and assess data; from Vannevar Bush how and why to employ these concepts; and from Abdel Omran a fertile new area in which to apply them. The accretion of scientific insights and tools created a geometric rise in scientific capacity that steepened slowly after Bacon, more rapidly during and after the industrial revolution, and exploded after the anamnestic boost from *Science, The Endless Frontier* [17]*.* There is no evidence of its slowing. Confirmatory evidence of the understanding of the importance of research science in economic and political arena can be seen in the congressional reaction to President Trump reducing all non-defense department research budgets. Congress brought 10% back to the agency request and increased the remaining 90% above the request [23].

This historical trajectory gives strength to the argument that reason and persistence can gain footholds in open societies and move the arcs of change. It also allows us to see our current status as both a logical emergence but also an unpredictable outcome. The improvements in the efficacy of clinical interventions over the past two generations have tumbled forth at lightning speed. However, the lesson history teaches is that going forward, we now know we must assume an accelerating trajectory and cost and, hence, plan accordingly.

From the perspective of population health, two pathways emerged from the post-World War II era to the present, the impact of one supporting the other. In 1948 the National Institutes of Health began multigeneration support for the Framingham Heart Study, one of America’s great medical gifts to the world. In 1961 the Framingham investigators published a paper identifying “factors of risk” that were predictive and causative of subsequent heart disease; these were smoking and hypertension [24].

While suspicions existed that these and other factors contributed to heart disease and other maladies, this was the first unassailable evidence. Over subsequent years the Framingham investigators and others added elevated lipids, obesity, and sedentarism to the list. Controlling these factors, knocking numbers back to new normals, was taken to be preventive. The medical establishment coalesced around an aggressive assault on risk factors. With the evidentiary report of the Surgeon General’s report on smoking in 1964 coupled with multiple confirmatory reports, blood pressure, cholesterol, and the prevalence of smoking all began to fall.

At the end of World War II clinical medicine had changed little from the end of the nineteenth century. In the late 1940s, a day in the hospital cost just over $10. Handholding and compassion were its chief non-surgical attributes. A heart attack patient was bed ridden for a month with no blood thinners, now clear-cut malpractice, and high-end care was being spoon fed so as to limit exertion. There was no imaging and no ability to limit the damage of the acute assault. Cardiac medications were two, digitalis from the eighteenth century and nitroglycerin from the 19^th^. The most effective diuretic was a mercury containing poison. But antibiotics were on the horizon; everything was about to change, and change fast. Every medical specialty was swept up in the waves of innovation.

Medical science has had three tectonic transformations since Vannevar Bush unleashed American scientific ingenuity. The first was the mechanical, physiological, and traditional biochemistry explosion which gave us, among other things, intensive care units, cardiovascular procedures and capacities, dialysis, and durable prosthetic joints. The second, overlapping the first, was unlocking the immune system which led to steroids, transplants, chemotherapy, and therapeutic insights into immune-system disorders. The third is the genetics revolution which has opened the floodgates to tiers of innovation and change that can barely be imagined. Inserting genes from other people, or species; correcting germ line genetic composition; identifying subsets of diseases that will each have their own specific therapies are just the beginning. Transformations yet to come, but on the horizon, are neurological and psychological interventions which even with today’s insights will be beyond current imagination. Facilitating this will be technologies non-existent a decade ago: artificial intelligence, “big data”, social networking, and computing power enhanced by orders of magnitude,

What do all these waves share? Aside from emerging from the insights of Sir Francis, amplified by those of Lind, Bush, Omran, and many others, what is the common denominator than binds them together into one profoundly important message for our time? They are all expensive. They are all very expensive. If the diseases were lined up in a column on a spread sheet next to their incidence and followed by the cost of therapy and its duration, ever expanding, the simple act of multiplication will give a minister of finance apoplexy.

That this is a likely outcome, at least in symbolic form, is bolstered by the observation that the world is fast approaching the milestone moment when every person on the planet will have a smart phone. Everyone will know what is available. The farmer in up country Ghana, the textile worker in Vietnam, the fisherman in a remote Bangladesh village, a Taliban zealot in Afghanistan, and an angry, self-reliant farmer in a “holler” in Kentucky will all want what is available for herself, his uncle, their child. What does the minister of finance do then? In addition, most advanced economies have a rapidly expanding population of older, retired people and, as fertility falls nearly everywhere [25], a rapidly diminishing population of young employed people whose taxes and productivity support their elders, further accentuating the fiscal burden of chronic illness. A perfect storm.

Over half of countries in Africa have dialysis for chronic end stage kidney disease [26]. Waiting list are long and nearly 60% of patients stop dialysis while it remains clinically indicated. Were all eligible patients to obtain necessary dialysis, the costs would consume between 15 and 55% of the government expenditure for health care, an unattainable goal [27]. While the contributory causes of kidney failure in most emerging economies are not as concentrated on hypertension and diabetes as they are in advanced economies, they are the most important causes [26]. However, because of severe budgetary constraints, poor countries are even more dependent upon prevention than advanced economies. In 2015, Latin America had the highest death rate from chronic kidney disease; in Mexico, with the highest regional rate, more than 50% were associated with diabetes [28]. Heart disease, stroke, and cancer have similar worldwide patterns and their fiscal and political impact is increasing. While mortality rates for many chronic diseases are decreasing, much of the decrease is attributable to increased longevity thus the lifespan fiscal impact increases. Is there a way out?

### The way out

The only path forward for public health is to assume a leadership role in adopting and promulgating public policies in the public square that are designed to prevent the emergence of risk factors, primordial prevention [5]. Data from large surveillance studies reveal that obesity and diabetes incidences are surging world-wide with well-known downstream impacts [6]. Since reversing these conditions on a population level is not doable, the primacy of prevention is only enhanced.

The first policy target for public health to engage is the obesity epidemic. The cost of the obesity cascade can approach 25% of a nation’s health care bill [29]. Of the top 5 leading causes of premature death and disability in the 50–74 age group, diabetes is the only disease that is increasing, and by a whopping 25%, since 1990 (2, p1212; panel E.) Kidney disease, thanks to diabetes, has climbed to number eight. A recent, and powerful, paper from RTI and the World Obesity Federation [30] calculates the current and future economic impact of obesity and overweight, concluding that by 2060 their fiscal impact will reach 3.29% of global gross domestic product. Initially astounding; upon reflection, hardly so. These data are convincing evidence that if therapy is the dominant approach to obesity or diabetes, the world loses. Most of it is preventable, in future generations.

#### Public health only rarely ventures into the public square

Its professional organizations attempt to influence political decision makers. It is not effective; it does not change consumer behavior [31]. Influencing policy makers requires money and public health organizations cannot compete with its corporate rivals. There needs to be a tectonic shift in how public health shapes policy. The current analyses of national or regional attempts to curb obesity, of which the recent study from Fiji [11] is of the highest caliber, do not tie the effort to the coming fiscal crises. There will be a demand for health care and an inability to deliver it. Ministries of finance and national or regional central banks need to be challenged and engaged. Public health organizations and institutions need to be much louder and reach a wider audience.

The corporate pushback has been and will be fierce [13], arguing that their freedom to market products desired by the public cannot be abridged, that their products satisfy public demand, that they make life in both emerging and advanced economies more satisfying and comfortable. And there will be some truths in these arguments.

The opposing truths will be that by promulgating disease emergence, they will impede long term development, divert needed resources to unnecessary health problems they help create, and distort national priorities in destabilizing ways. Creating effective arguments will take time, political skills, public engagement, and tenacity. Public health will need to be visible, loud, persistent, and persuasive.

The global and, in particular, American corporate culture is alive and well. It possesses an enormous capacity for innovation and resilience. The global entrepreneur is adept, agile, imaginative, creative, and clever. As they are persuaded, pushed, and coerced to change, there will surely be winners and losers; there always are. But there will be more winners than losers; there always are. New industries, new products, and new ideas will emerge; they always do. There will be more consumers for more products; there always are.

Should there be any doubt about corporate innovation, flipping through any month’s issue of *Science,* the leading U.S. science journal, will squelch any concern. From the James Webb telescope to aerial surveillance to collect sophisticated agricultural and environmental data to CRISPR technology to the rapid development of mRNA vaccines for COVID 19, corporate opportunities emerge even before the recent ones have had time to set. The global food and drink companies are just as nimble.

A brief survey of the obvious can serve as a window into this arena. The overriding goal is quite simple. It is to make available and promulgate affordable, healthy, tasty, culturally desirable food, to the disadvantaged sectors of society. Trade, television, and subsidies are early targets. Trade agreements, particularly regional structures that are not bound to World Trade Organization regulations, are heavily tilted in favor of the wealthy partner [32]. As urbanization accelerates in emerging economies, both husband and wife join the workforce, their fewer children attend school, and family life becomes frenetic and full. The multinational food corporations offer rescue with tasty, safe, long-shelf-life foods that kids love. All true; all important. But the outcome is obesity. Surely, desirable and tasty food without the fat, salt, and calories can be created. A tax on these components, designed not to force a new eating pattern by a patronizing, all knowing state, but levied to help defray future medical costs, will be a stimulus for both sides of the transaction to change. The commercial determinants of health are powerful [13]. Relying on good will and corporate social conscience will not work; across the board policies will work. It can be done. Chile launched a “war on obesity” and needed a decade to get a law passed. However, it has had an impact in changing the purchasing patterns of obesogenic foods and drinks for children [33, 34]. Clinical endpoints will only emerge later and only if the regulations are kept intact.

When emerging countries face unmeetable demands for health care that are recognized as diet related, the accusation will be that the trade arrangements designed by wealthy countries were really the importation of diabetes, heart disease, and cancer. While not a comfortable diplomatic place to be, the political realities in both manufacturing and recipient countries pose formidable barriers to instituting change.

Tied to diet and obesity is TV advertising to children. In most markets over 50% of TV ads during children’s programs is for high fat, high sugar, high caloric foods. As has been demonstrated with health messaging embedded in Sesame Street, young children can not only alter behavior, but carry the message to their parents [35].

Another way to influence children’s health is utilizing conditional cash transfers. In multiple Latin American countries, checks sent to mothers—always mothers—if the children’s school attendance or vaccination rates meet prespecified levels does significantly boost adherence [36]. Would there be a change in U.S. food consumption, and in what is available in local markets, if checks were sent for children (from poor families) who are normal weight for height? This is an easy enough hypothesis to test.

Crop subsidies can be used to foster good health. The U.S. should get rid of the corn subsidy, at least that portion used for fructose syrup. The first step should be to move the Iowa presidential caucus to May so that all candidates of all parties do not have to swear allegiance to corn in the first step of a presidential run. The European Union agricultural subsidies push a lot of unwanted high calorie dairy products to developing economies. The trade world needs to incorporate health as one of its key metrics; the public health community can now volunteer to serve on advisory committees to the U.S. trade representative [37].

This sampling serves only to introduce the broad array of possibilities.

## Conclusion

Because of its fiscal burden, health care has or will become a lightning rod for political attention around the world. Without the innovative input of public health, the solutions are likely to be dreadful, aggravating class-based levels of access, increasing societal antagonisms, and raising global tensions. There is a time to act; it is now.

## Data Availability

NA. No data beyond what is extracted from citations is used.

## Declarations

### Author information

The author has been astonished in his 20 years of writing on the centrality of chronic diseases and their impact by the lack of interest by academic and “public” public health. This lacuna is made even more pronounced by the increasing availability of irrefutable data.
